# The prevalence and risk factors of posttraumatic cerebral infarction in patients with traumatic brain injury: a systematic review and meta-analysis

**DOI:** 10.1080/21655979.2022.2070999

**Published:** 2022-05-06

**Authors:** Chen Zhi-Ling, Li Qi, Yang Jun-Yong, Yuan Bang-Qing

**Affiliations:** The 900th Hospital of the Chinese People`s Liberation Army Joint Logistic Support Force, Fuzhou, China

**Keywords:** Posttraumatic cerebral infarction, PTCI, traumatic brain injury, TBI, prevalence, risk factors, meta-analysis

## Abstract

Posttraumatic cerebral infarction (PTCI) is a serious complication of traumatic brain injury (TBI), and the prevalence and risk factors of PTCI in TBI patients are in dispute. We systematically searched the literature in the PubMed, Embase, and Cochrane library up to October 2021 to identify studies on the prevalence and risk factors of PTCI in patients with TBI. The quality of observational studies was assessed by the Newcastle–Ottawa scale tool. Random-effects model was conducted. The Higgins` *I*^2^ statistic was used to measure heterogeneity between trials. Moreover, sensitive analyses were conducted to assess whether the pooled result was credible and robust. Eleven studies (3696 total TBI patients) were included. The pooled prevalence of PTCI in TBI patients was 14% (95% CI, 0.11–0.17; I^2^ = 83.1%). Sensitive analyses showed that the pooled prevalence of PTCI was 13% (95% CI, 0.10–0.15; I^2^ = 69.2%) by omitting Su et al. The prevalence of PTCI was associated with a lower Glasgow Coma Scale (GCS) score (OR, 0.33; 95% CI, 0.14–0.77; I^2^ = 99.2%), pupillary dilation (OR, 4.73; 95% CI, 4.30–5.19; I^2^ = 85.6%), abnormal PT (OR, 1.16; 95% CI,1.05–2.47; I^2^ = 99.2%), hematoma location (OR, 1.16; 95% CI,1.05–2.47; I^2^ = 99.2%) and hematoma volume (OR, 1.16; 95% CI,1.05–2.47; I^2^ = 99.2%). Whereas hypotensive shock, duraplasty, cerebral herniation, and thrombocytopenia were not statistically associated with PTCI. Lower GCS, pupillary dilation, abnormal PT, hematoma location, and hematoma volume were risk factors for PTCI. Considering some limitations, the conclusion of our study should be interpreted with caution.

## graphical abstract

**Figure:**
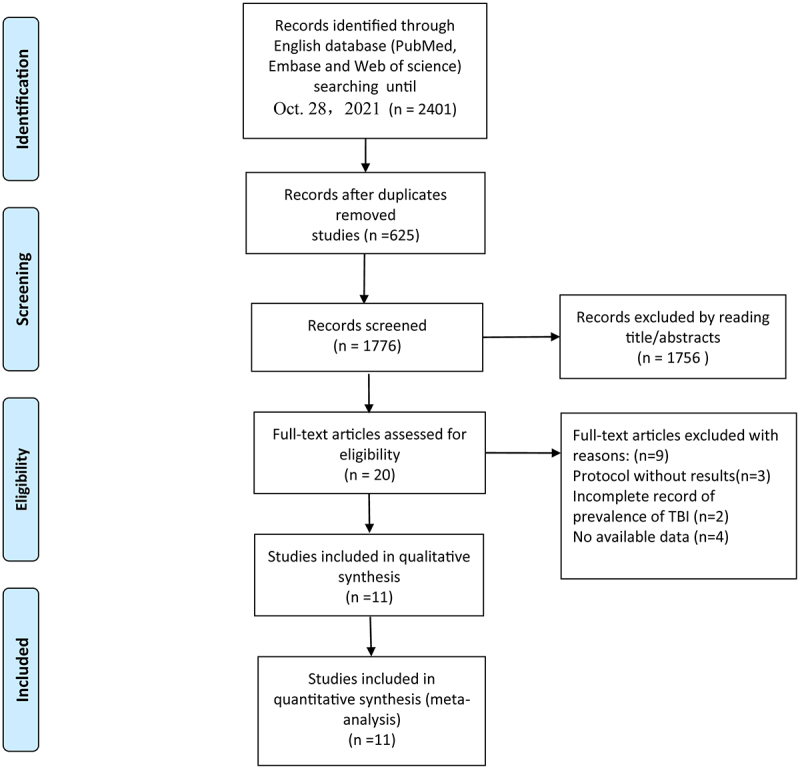

## Hightlight

- The pooled prevalence of PTCI in patients with TBI was approximately 13%.
- Lower Glasgow Coma Scale (GCS) score, pupillary dilation, abnormal PT, hematoma location, and hematoma volume were independent risk factors of PTCI in patients with TBI.
- Hypotensive shock, duraplasty, cerebral herniation, and thrombocytopenia may be potential risk factors for PTCI in patients with TBI.

## Introduction

Traumatic brain injury (TBI) is an injury of cerebral morphology caused by direct and indirect violence, with characteristics: symptoms and signs of apparent acute illness and rapid development, and high deformity and mortality rates [1]. The morbidity of brain injury was reported to be the first in trauma, range from 9% to 21% [2]. In the United States, brain injury is ranked first in the traumatic death and accounted for about a third to a half of the traumatic death toll [3]. 23,500 hospitalizations, 50,000 deaths, and 70,000 permanent disabilities annually were associated with TBI according to the state-based administrative health-care data during 2007 and 2013[4]. That reducing the incidence and improving the prognosis of TBI is one of the burning issues.

Posttraumatic cerebral infarction (PTCI) is a common and serious complication of TBI, with an incidence range from 1.9% to 16.67 and a high mortality of 75% [5–7]. The incidence of PTCI following TBI is still in dispute. Focal mass effect and vascular impingement caused by Cerebral herniation, cerebral vasospasm, thromboembolism, venous congestion at craniectomy sites or cerebrovascular injury were reported to be the pathological mechanism of PTCI [6,8]. However, the exact pathogenesis of PTCI is still uncertain and effective treatments are lacking.

This current meta-analysis and systematic review aimed to systematically investigate the prevalence of PTCI and related risk factors in patients with traumatic brain injury, which may contribute to dispel the aforementioned controversy and uncertainty and provide related optimum epidemiological evidence.

## Methods

The current systematic review and meta-analysis was performed according to the Guidelines for Meta-analysis of Observational Studies in Epidemiology (MOOSE) [9] and the Preferred Reporting Items for Systematic reviews and Meta-Analysis (PRISMA) guidelines [10]. This meta-analysis was conducted by two independent researchers (CZL and LQ) and a third reviewer (YBQ) was consulted to reach a consensus if disagreements were emerged.

## Literature search

Systematic searches of the PubMed, Embase, and Web of science from January 2005 to 28, October 2021 were conducted to identify studies that reported the prevalence and risk factors for PTCI in patients with TBI. The search strategy was conducted using the terms of ‘brain trauma, Traimatic’, ‘Traumatic brain injury’, ‘Cerebral infarction’ and their variants. Also, we manually checked the references of the included studies and some important reviews for any potential inclusion. Non-English publications were excluded.

## Inclusion criteria

In the current meta-analysis, studies met the following inclusion criteria: (1) researches published between January 2005 and October 2021; (2) studies reported the prevalence or risk factors for PTCI in patients with TBI; (3) definitive diagnosis cerebral infarction was reported; (4) odds ratios (OR) or risk ratio (RR) or adjusted OR or RR and the corresponding 95% confidence interval (CI) were provided; (5) studies published in full-text form and English. Meanwhile, related letters, comments, and review articles were excluded.

## Data extraction and assessment of quality

We used a pre-designed data collection form to extract the following study data: last name of the first author, publication year, country, study design, study period, case number of patients with TBI, age and sex ratio of patients with TBI, case number of patients with PTCI, prevalence of PTCI, diagnostic tools of PTCI, risk factors for PTCI, and the quality of the included studies. Besides, the primary outcome is the prevalence of PTCI in patients with TBI. And the secondary outcome is the related risk factors for PTCI in patients with TBI. Only ORs or RRs with 95% CIs on the multivariate analysis of included studies were extracted.

Newcastle-Ottawa Scale (NOS) [11] was used to assess the quality of case-control and cohort studies. The NOS contains three dimensions, including selection of participants, comparability of study groups, and ascertainment of outcome or exposure. The NOS score ranged from 0 to 9, and studies with scores ≥7 were considered to be of high quality and NOS < 7 were considered moderate-quality.

## Statistical analysis

Data on the prevalence of PTCI following TBI were extracted from included studies, and a random-effects model and the generic inverse-variance were used to pool the prevalence with 95% CI. And a random effect model was conducted to calculate the ORs and 95% CI of the correlative dimension of risk factors for PTCI in patients with TBI. The same risk factor was reported in two or more studies was pooled in this meta-analysis. Statistical heterogeneity was quantified using Cochran’s Q test and I2 statistic, and we considered significant heterogeneity if the I2 > 50% or p value of Cochran’s Q test was less than 0.05^12^. Studies were pooled using random-effects models if high heterogeneity among studies was observed. Moreover, subgroup analysis was conducted to explore potential heterogeneity among the included studies, including NOS score, article design, sample size, diagnostic modality, and study location. Sensitivity analysis was conducted to explore the potential heterogeneity by omitting the studies for each analysis. Publication bias was assessed by visual inspection of a funnel plot. Further, Egger’s regression quantitatively and Begg-Mazumdar rank continuity correlation were conducted to explore the potential publication bias. Two-side p < 0.05 was considered as statistical significance. All the above statistical analyses were performed with Stata 12.0 (Stata Corporation, College Station, TX, USA).

## Result

### Brief introduction

This current meta-analysis was conducted to explore the prevalence and risk factors of PTCI in TBI patients. Literature was systematically searched in the PubMed, Embase, and Cochrane library to identify studies reporting the prevalence and risk factors of PTCI in patients with TBI. Total 11 studies including 3696 TBI patients were included in the meta-analysis. The pooled prevalence of PTCI in TBI patients was 13% (95% CI, 0.10–0.15; I^2^ = 69.2%). Lower GCS, pupillary dilation, abnormal PT, hematoma location, and hematoma volume were risk factors for PTCI.

## Study selection and characteristics

**Figure 1.**
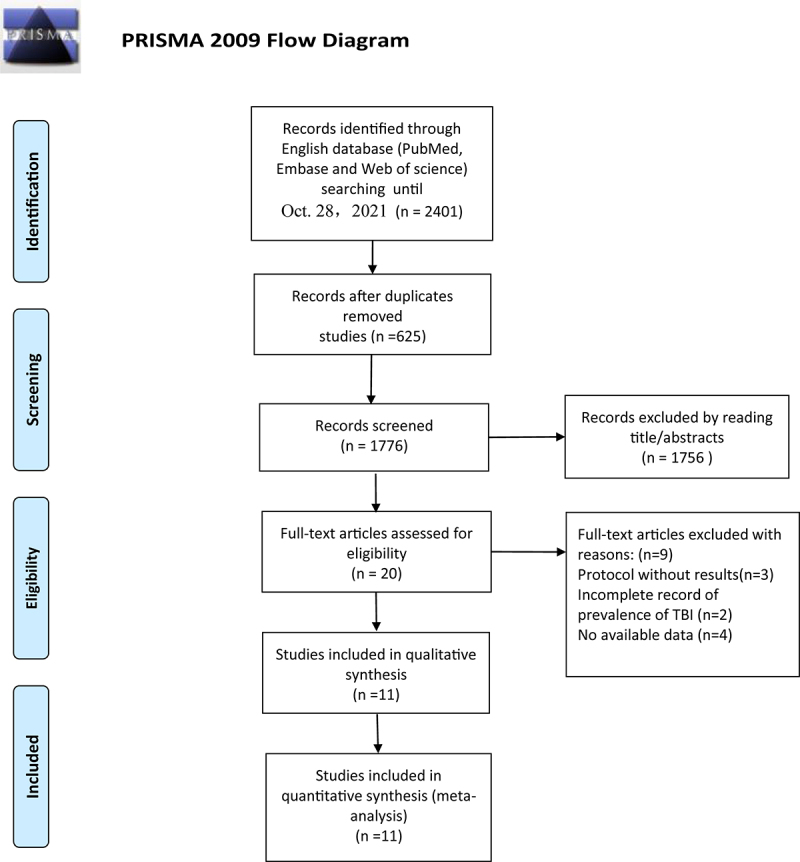
PRISMA flow diagram of literature selection for this meta-analysis.

In this meta-analysis, 2401 literature citations were searched from an electronic database. After removing duplicates and review of titles and abstract, 20 articles were evaluated by reading the full text and checking reference lists. From the secondary screening, nine studies were excluded due to protocol; without results (n = 3), incomplete record of prevalence of TBI (n = 2) and no available data on risk factors (n = 4). Ultimately, 11 studies [5,7,12–20] were deemed suitable to be included based on inclusion criteria. Eleven studies had reported the prevalence of PTCI in patients with TBI and related risk factors for PPTCI (Figure 1).

A total of 3696 patients with TBI in 11 studies were included in this meta-analysis, which was published between 2006 and 2021. All the studies were observational research in which nine studies were retrospective study and one studies were prospective study, except for one randomized controlled study (RCT)[14]. These involved patients with mean age ranging from 4 months old to 90 years old and the sample size of the included studies was from 89 to 1431. Seven studies were conducted in China, one each in Germany, Mexico, and Italy, and one study was performed in 10 hospitals in the UK and 4 in Malaysia. The diagnostic tool of PTCI in four studies was computed tomography (CT) and in seven studies was combination CT with magnetic resonance imaging (MRI). Baseline characteristics of these included studies are summarized in Table 1.

**Table 1.** Characteristics of the included studies in the final analysis

Author (et.al), Year [1]	Country	Study design	Period of research	Patients with TBI (n)	Age (years)[2]	Sex ratio (M/ F)	Patients with PTCI (n)		Prevalence of PTCI(%)	Diagnostic tool of PTCI	Risk factors for TBI	NOS score
Wu2021 [18]	China	Retrospective cohort study	January 2019 and September 2020	297	37.4 (0.9 ~ 90)	190/107	32	10.77		CT	low admission, skull base fractures, traumatic SAH, brain herniation, hypotensive shock, and decompressive craniectomy	7
Mehdi2021 [20]	Germany	prospective cohort study	July 2017 and May 2020	130	74.5(IQR: 28)	78/52	14	10.8		CT	brain natriuretic peptide, Pupils (medium-sized and reactive)	8
Mahmood2021[14]	10 hospitals in the UK and 4 in Malaysia	randomized controlled study	February 2013 and January 2019	1431	45 (29 ~ 63)	1413/354	159	11.0		CT	tranexamic acid5,7,12–20	9
Su2018[16]	Taiwan, China	Retrospective cohort study	2007 to 2012	173	50.5 ± 18.5	126/47	54	31.2		CT	preoperative GCS score, pupillary dilation, subdural hematoma and craniectomy size	6
Zhang2016[19]	China	Retrospective cohort study	June 2009 to March 2014	88	30.3(4 months to 67 years)	72/16	9	10.2		CT	the hematoma location, volume, the largest thickness and mid-line shift, basal cisterns compression, traumatic subarachnoid hemorrhage, pupil dilatation, pre-operative GCS score, ΔGCS and intraoperative brain pressure	6
Liu2015[13]	China	Retrospective cohort study	2008 to 2013	339	40 (21–51)	282/57	69	20.4		CT+MRI	hyperthermia in the first 24 h, thrombocytopenia, abnormal prothrombin time and traumatic subarachnoid hemorrhage	6
Wang2014[17]	China	Retrospective cohort study	January 2005 to January 2012	176	38.8 (2–70)	124/52	32	18.2		CT+MRI	Hematoma location, Duration of preoperative brain hernia, GCS, Hematoma volume, Mydriasis, Occurrence of preoperative shock	7
Chen2013[12]	China	Retrospective cohort study	January 2005 to December 2010	265	32.4 (13–63)	155/110	28	10.57		CT+MRI	the thrombocytopenia, abnormal PT, D-dimer (>2 mg/L),or DIC scores	7
Tian2008[5]	China	Retrospective cohort study	January 2004 to December 2005	353	31.2 (2–86)	213/140	42	11.9		CT	Poor admission GCS, low systolic BP, brain herniation, and decompression craniotomy	7
Tawil2008[7]	Mexico	Retrospective cohort study	January 2004 through December 2005	355	36 (11–90)	288/67	31	8.7		CT+MRI	the presence of blunt cerebral vascular injury, the need for crani otomy or treatment with recombinant factor VIIa	5
Marino2006[15]	Italy	Retrospective cohort study	June 1998 and November 2001	89	34.4 ± 17.6	75/14	17	19.1		CT	age, sex, severity of brain trauma, and time spent in ICU	6

TBI: traumatic brain injury; PTCI: posttraumatic cerebral infarction; CT:Computerized tomography; MRI: Magnetic resonance imaging; SAH: subarachnoid hemorrhage; NA: not available; GCS: Glasgow Coma Scale; LOS: hospital length of stay; PT: prothrombin time; DIC: disseminated intravascular coagulation; BP: blood pressure; ICU: Intensive care unit; NOS: Newcastle–Ottawa Scale. [1]Reference; [2] Data were shown as mean ± standard deviation (SD) or Median (IQR);

The risk of bias was assessed using the Newcastle–Ottawa scale tool, and the details were summarized in Table S1. The score of five studies [7,13,15,16,19] less than 7 were judged at ‘high risk of bias,’ while six studies [5,12,14,17,18,20,21] with score equivalent to or more than 7 were classified as ‘low risk of bias.’ No study was excluded due to methodological quality.

## Meta-analyses of the prevalence of PTCI following TBI

**Figure 2.**
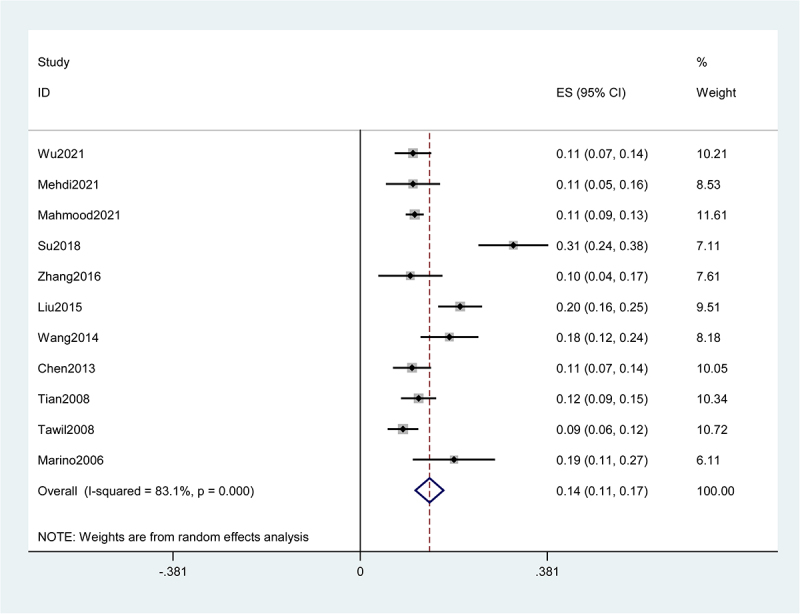
Forest plot for prevalence of PTCI in patients with TBI using random-effects mode.

Eleven studies involving 3696 patients with TBI reported the incidence of PTCI, ranging from 8.7% to 31.2%. The synthetic result of 11 eligible studies showed that the pooled incidence of PTCI in patients with TBI was 14% (95% CI = 11–17%) using random effects analysis, with high heterogeneity (I2 = 83.1%, P < 0.01) (Figure 2).

Subgroup analyses were performed based on NOS score, article design, sample size, diagnostic tool, and study location. Subgroup analyses based on diagnostic tool showed that studies with sample size≥ 300 (13%), studies with sample size 300 (15%), studies using CT as a measuring tool (14%), studies using CT combined with MRI (14%), studies conducted in China (16%) and non-China (14%) were similar to the pooled prevalence in this meta-analysis. Six studies with NOS ≥ 7 reported the incidence of PTCI was 18% (95% CI = 10–26%), with high interstudy heterogeneity (I2 = 91.6%; p < 0.001). Meanwhile, five studies with NOS 7 reported the rate of sarcopenia was 12% (95% CI = 11–17%), with low interstudy heterogeneity (I2 = 16.9%; p = 0.305). According to article design, nine retrospective studies reported that the incidence of PTCI was 15% (95% CI = 11–19%), with high interstudy heterogeneity (I2 = 85.7%; p < 0.001), while two prospective studies reported that the incidence of PTCI was 11% (95% CI = 10–13%), with no heterogeneity (I2 = 0; p = 0.904). (Table S2)

**Figure 3.**
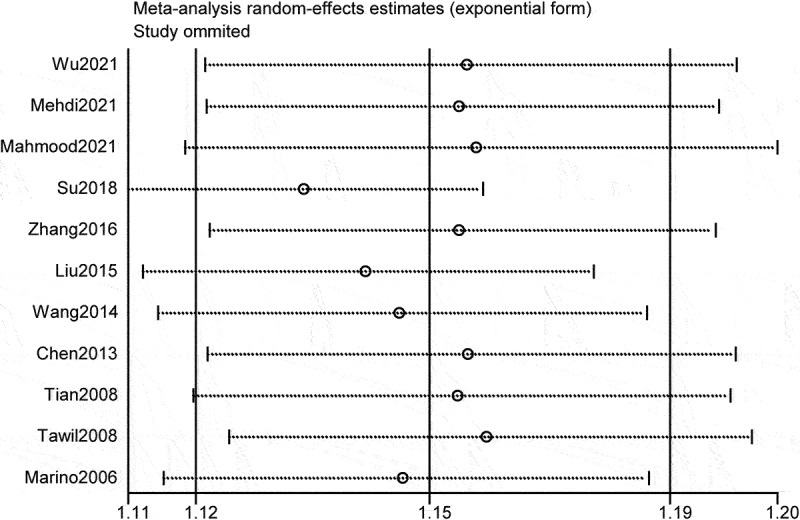
Sensitivity analysis for prevalence of PTCI in patients with TBI in the meta-analysis.

**Figure 4.**
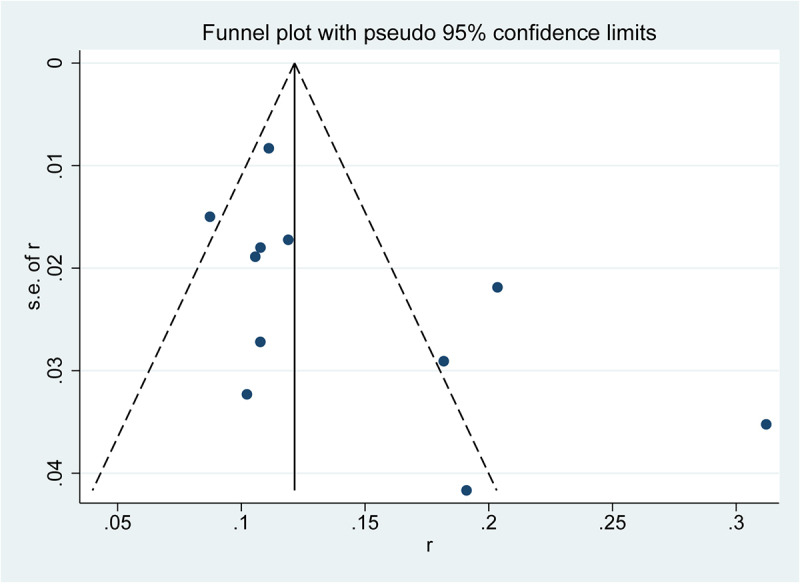
Funnel plots for prevalence of PTCI in patients with TBI in the meta-analysis.

**Figure 5.**
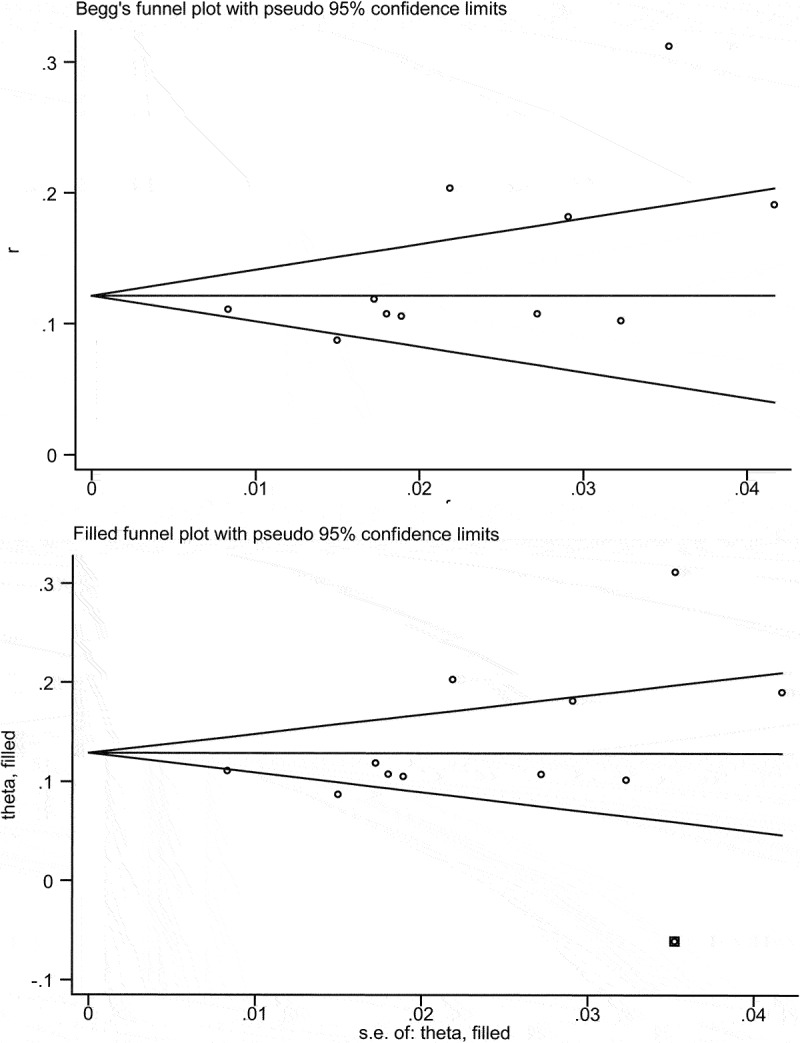
Trim and filling for prevalence of PTCI in patients with TBI in the meta-analysis.

Besides, sensitivity analysis was conducted to assess the presence of substantial heterogeneity of the pooled results and explore the source of heterogeneity. The results of sensitivity analyses showed that Su et al. [16] were the highest source of heterogeneity, which was the prime determinants of the pooled incidence of PTCI Figure 3). After eliminating Su et al., the pooled incidence of PTCI was 13% (95% CI = 10–15%; I2 = 69.2; p = 0.001) (Figure S1). Meanwhile, the publication bias was visually recognized using the Doi plot of funnel plot, and minor asymmetry was inspected (Figure 4). Further Begg’s and Egger’s tests were performed (Begg: p = 0.043, Egger: P = 0.073), which indicated the existence of potential publication bias. However, the pooled prevalence was basically unchanged according to the results of Trimming estimator and Filled analyses. (Figure 5)

## Meta-analyses of risk factors for PTCI following TBI

Eleven studies reported risk factors for the incidence of PTCI in patients with TBI and 10 potential risk factors for PTCI were assessed in this meta-analysis. The details of extracting risk factors for PTCI was showed in Table S3. ORs or RRs and 95% CI of multivariate analysis were extracted and pooled using a random effects model. The pooled results showed that the prevalence of PTCI was statistically significantly associated with six factors: pupillary dilation (OR = 4.17, 95% CI = 1.92–9.06), traumatic subarachnoid hemorrhage (SAH) (OR = 3.51, 95% CI = 1.41–8.72), abnormal prothrombin time (PT) (OR = 5.01, 95% CI = 3.23–7.77), hematoma location (OR = 20.27, 95% CI = 5.24–78.42), and hematoma volume (OR = 8.07, 95% CI = 2.86–22.76), while lower Glasgow Coma Scale (GCS) score (OR, 0.33; 95% CI, 0.14–0.77; I^2^ = 99.2%) is a protected risk for PTCI. Moreover, hypotensive shock (OR = 1.23, 95% CI = 0.30–5.00), duraplasty (OR = 1.28, 95% CI = 0.66–2.47), cerebral herniation (OR = 1.82, 95% CI = 0.39–8.59), and thrombocytopenia (OR = 5.83, 95% CI = 0.80–42.69) were most likely not risk factors according to the pooled result in this meta-analysis (Table 2) (Figure S2).

**Table 2.** Risk factors for PTCI in patients with TBI in this meta-analysis

	Number oftrials	Pooled OR(95% CI)	I2 (%)
hypotensive shock	4	1.23(0.30 ~ 5.00)	94.5
duraplasty	4	1.28(0.66 ~ 2.47)	81.4
GCS	3	0.33(0.14 ~ 0.77)	63.8
brain herniation	3	1.82(0.39 ~ 8.59)	90.8
pupillary dilation	2	4.17(1.92 ~ 9.06)	0.0
traumatic SAH	2	3.51(1.41 ~ 8.72)	65.9
thrombocytopenia	2	5.83(0.80 ~ 42.69)	88.6
abnormal PT	2	5.01(3.23 ~ 7.77)	0.0
hematoma location	2	20.27(5.24 ~ 78.42)	0.0
hematoma volume	2	8.07(2.86 ~ 22.76)	0.0

PTCI = Posttraumatic cerebral infarction; OR = odds rate; CI = confident interval; GCS: Glasgow Coma Scale; SAH: subarachnoid hemorrhage; PT: prothrombin time.

## Discussion

According to previous studies, the incidence of PTCI ranged from 1.9% to 16.67^5-^[7]. In this current meta-analysis, the pooled prevalence of PTCI was 14% (95%CI = 11–17%), with high heterogeneity (I2 = 83.1%; p < 0.001). After eliminating Su et al. which was the highest source of heterogeneity, the pooled incidence of PTCI was 13% (95% CI = 10–15%). In 1990, Mirvis et al. [8] first reported the incidence of PTCI following TBI and the diagnostic rate of PTCI in 22 patients with TBI was 1.9% using CT. With the development of CT and MRI, the diagnostic rate of PTCI significantly increased. Previous studies suggested that computed tomography angiography (CTA) was useful in the early diagnosis of PTCI, which was widely used for diagnosing cerebrovascular disease [22,23]. Besides, clinicians and radiologists realized the severity of PTCI and paid more attention to PTCI.

In addition, the prevalence of PTCI following TBI in different classifications of severity is inconsistent. Studies reported that the incidence of PTCI in patients with a GCS score of 5 or less was significantly higher than in patients with a GCS score of higher than 5^5,^ [7,24]. Similarly, Su et al. reported the prevalence of PTCI in 173 patients with moderate or serious TBI who underwent decompressive craniectomy (DC), and the result showed that the incidence of post-DC PTCI was significantly higher than other research [16]. Hence, the severity of TBI and diagnostic tool were important factors for the prevalence of PTCI.

Moreover, we further explored the potential risk factors for PTCI in patients with TBI and several risk factors for the occurrence of PTCI were identified, including pupillary dilation, abnormal PT, hematoma location, and hematoma volume. Post-traumatic cerebral infarction is a cerebrovascular disease caused by violence-induced injuries of the brain, which leads to ischemic necrosis of corresponding brain tissue [25]. The main cause of PTCI following TBI was the ischemia and hypoxia of brain tissue, and the severity and duration of the ischemia and hypoxia were closely related to PTCI [26].

Glasgow coma scale (GCS) was widely used to assess the status and prognosis of the brain disease. Severe brain injuries, such as diffuse subarachnoid hemorrhage, extensive brain contusion and laceration and malignant brain swelling, can lead to a sharp increase in intracranial pressure, and even the formation of cerebral hernia, resulting in compression, displacement, or distortion of cerebral vessels, resulting in cerebral ischemic injury and cerebral infarction. Besides, patients with lower GCS, especially those accompanied by cerebral herniation, were highly susceptible to PTCI because of insufficient cerebral perfusion pressure and obstruction of cerebral arteries [15]. Hence, cerebral herniation and pupillary dilation were supposed to be related to PTCI, but the correlation between Cerebral herniation and PTCI was not statistically significant in this meta-analysis. Cerebral herniation was reported to be a risk factor for PTCI in multiple studies [5,6,17,18,27]. Brian hernia was developed by the brain tissue displacement caused by depressed fracture, intracranial hematoma, and encephaledema, and further led to ischemia and hypoxia of brain tissue.

Traumatic subarachnoid hemorrhage (tSAH) was considered as an important factor for morbidity and mortality of PTCI with a high prevalence of 33–60% [28]. On the one hand, tSAH can lead to cerebral vasospasm caused by the pyrolysis products of local hematocele and stimulation of various chemical factors. On the other hand, tSAH can catalyze oxidation and radical reaction, result in the damage to lipid peroxidation, lead to the alteration of membrane permeability, and cause cell death and dissolution of brain tissue [6,28,29]. And the hematoma location and volume were also the risk factors for PTCI. Moreover, patients with thrombocytopenia, abnormal prothrombin time, and blood coagulation dysfunction were prone to suffer from cerebral hemorrhage and PTCI.

Decompressive craniectomy (DC) and duraplasty were widely used in patients with serious intracranial pressure (ICP) caused by severe brain injury [30]. And DC and duraplasty were recommended as second-line treatment for encephaledema by the European Brain Injury Association (EBIC) and American Brain Injury Association (ABIC) guidelines [31]. Multiple researchers suggested DC was a preferred recommendation in patients with encephaledema caused by severe brain injury accompanied by extensive brain contusion, intracranial hematoma, or subdural hematoma [32–38]. Nevertheless, Caro et al. found that the incidence of PTCI was not reduced by DC in patients with acute mass subdural or intracerebral hematoma [39]. And Tamaki et al. even showed that the rapid decreases in ICP and the deterioration of hemorheology caused by rapid evacuation of intracerebral through DC increased the prevalence of PTCI [40]. Progressive removal of intracranial hematoma, decreased intracranial pressure, and stable cerebral perfusion pressure were important in the treatment of patients with serious encephaledema after TBI. Besides, previous studies deemed that old age, diabetes mellitus, and renal disease were risk factors for PTCI, which remained to be further explored.

To our knowledge, this was the first and comprehensive meta-analysis estimating the prevalence and risk factors for PTCI in patients with TBI so far. A large sample size from 11 studies with 3696 patients with TBI. To some extent, the resource of statistical heterogeneity among studies might be the different severity of TBI, different diagnostic tools, different population regions. Several potential limitations still exist in the current meta-analysis. First, included studies in this review contained patients with TBI vary in degree and further subgroup was conducted according to the severity of TBI. This is the reason that the incidence of PTCI was 31% in Su et al., which included patients with serious TBI and the incidence of PTCI was much higher than other studies. Second, most included studies were conducted in China, which may affect the overall conclusion. In addition, more risk factors were reported, but the association between them and PTCI cannot be explored because of the short of evidence. Finally, most of the studies included in this meta-analysis were retrospective, and subgroup analyses indicated that there was a potential bias. Hence, a random effect model was used to estimate the prevalence and risk factors for PTCI because of the high heterogeneity between included studies, and additional large-scale studies should be carried out to achieve a more comprehensive result of the prevalence and risk factors of PTCI in patients with TBI.

## Conclusion

In conclusion, PTCI was a serious complication of TBI with poor prognosis. Therefore, early diagnosis and therapy were vital. Higher GCS score, pupillary dilation, abnormal PT, hematoma location, and hematoma volume were risk factors for the prevalence of PTCI, while hypotensive shock, duraplasty, cerebral herniation, and thrombocytopenia were not associated with PTCI. Further high-quality trails should be carried out to explore potential risk factors for PTCI.

## Supplementary Material

Supplemental Material

## Data Availability

All data generated or analyzed during this study are included in this published article.

## Abbreviations

PTCI: Posttraumatic cerebral infarction

TBI: Traumatic brain injury

CI: Confidence interval

OR: odds ratio.
