# Supplementary material for: The prevalence and risk factors of posttraumatic cerebral infarction in patients with traumatic brain injury: a systematic review and meta-analysis
Source: Bioengineered. 2022 May 6;13(5):11706–17. doi: 10.1080/21655979.2022.2070999 (PMC9275913; doi:10.1080/21655979.2022.2070999)
Supplement: Supplemental Material [file KBIE_A_2070999_SM5651.zip › supplementary/Table_S1.docx]

| Table S1: Methodological quality of included studies based on the Newcastle–Ottawa Scale* | | | | |
| --- | --- | --- | --- | --- |
| studies (n=10) | Selection(0-4stars) | Comparability (0-2 stars) | Outcome (0-3 stars) | Total NOS score (0-9） |
| Wu2021 | **** | * | ** | 7 |
| Mehdi2021 | **** | ** | ** | 8 |
| Mahmood2021 | **** | ** | *** | 9 |
| Su2018 | ** | * | *** | 6 |
| Zhang2016 | ** | ** | ** | 6 |
| Liu2015 | *** | ** | ** | 6 |
| Wang2014 | *** | ** | ** | 7 |
| Chen2013 | *** | ** | ** | 7 |
| Tian2008 | **** | * | ** | 7 |
| Tawil2008 | ** | ** | * | 5 |
| Marino2006 | ** | ** | ** | 6 |
| *A study can be awarded a maximum of one star for each numbered item within the Selection and Exposure categories and maximum of two stars can be given for comparability. 1 A cohort study with a follow-up time > 6 months was awarded one star. 2 A cohort study with a follow-up rate > 75% was awarded one star | | | | |
