# Supplementary material for: The prevalence and risk factors of posttraumatic cerebral infarction in patients with traumatic brain injury: a systematic review and meta-analysis
Source: Bioengineered. 2022 May 6;13(5):11706–17. doi: 10.1080/21655979.2022.2070999 (PMC9275913; doi:10.1080/21655979.2022.2070999)
Supplement: Supplemental Material [file KBIE_A_2070999_SM5651.zip › supplementary/Table_S2.docx]

| **Table S2:** Subgroup analysis of prevalence of PTCI based on various factors. | | | | | | |
| --- | --- | --- | --- | --- | --- | --- |
| Outcomes | | Number of trials | OR/RR (95% CI) | | Heterogeneity, I2 (%) | |
| Pooled results | | 11 | 0.14(0.11-0.17) | | 83.1 | |
| Subgroup analyses based NOS score | | | | | | |
| NOS≥7 | | 6 | 0.12(0.11-0.17) | | 16.9 | |
| NOS˂7 | | 5 | 0.18(0.10-0.26) | | 91.6 | |
| Subgroup analyses based on article type | | | | | | |
| Retrospective study | | 9 | 0.15 (0.11-0.19) | | 85.7 | |
| Prospective study | | 2 | 0.11 (0.10-0.13) | | 0 | |
| Subgroup analyses based on number of sample | | | | | | |
| N≥300 | | 4 | 0.13 (0.09-0.16) | | 85.0 | |
| N˂300 | | 7 | 0.15 (0.11-0.17) | | 83.5 | |
| Subgroup analyses based on diagnostic tool | | | | | | |
| CT | | 4 | 0.14 (0.09-0.20) | | 87.5 | |
| CT+MRI | | 7 | 0.14 (0.10-0.18) | | 82.7 | |
| Subgroup analyses based on region | | | | | | |
| China | | 7 | 0.16 (0.11-0.20) | | 86.3 | |
| Other country | | 4 | 0.14 (0.11-0.17) | | 50.1 | |
| PTCI= Posttraumatic cerebral infarction; OR= odds ratio; RR= relative ratio; CI= confidence interval; CT:Computerized tomography; MRI: Magnetic resonance imaging; NOS= Newcastle-Ottawa Scale; | | | | | | |
|  |  | | |  | |  |
