# Supplementary figures and images for: The prevalence and risk factors of posttraumatic cerebral infarction in patients with traumatic brain injury: a systematic review and meta-analysis
Source: Bioengineered. 2022 May 6;13(5):11706–17. doi: 10.1080/21655979.2022.2070999 (PMC9275913; doi:10.1080/21655979.2022.2070999)

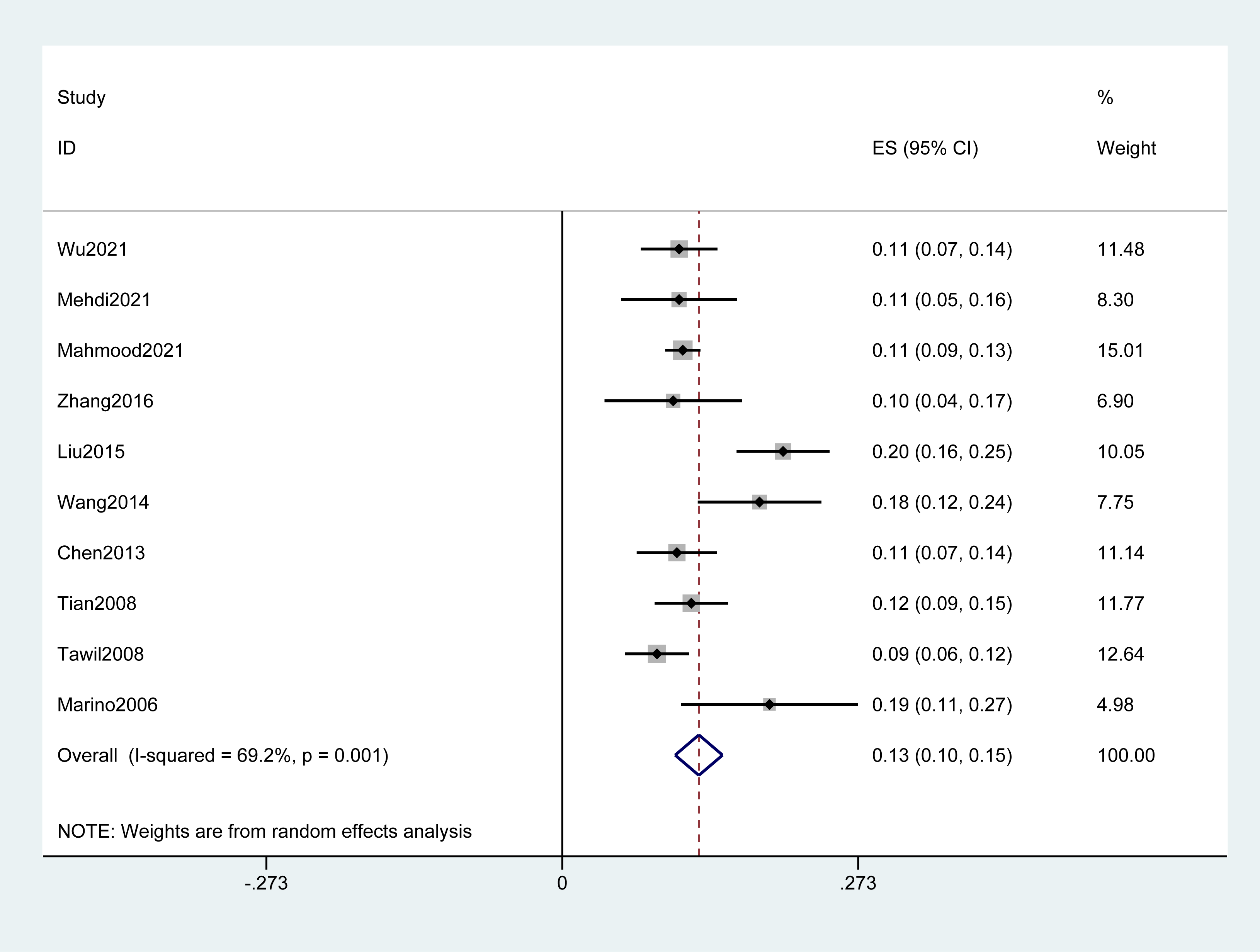

Supplement: Supplemental Material [file KBIE_A_2070999_SM5651.zip › supplementary/Figure S1.tif]

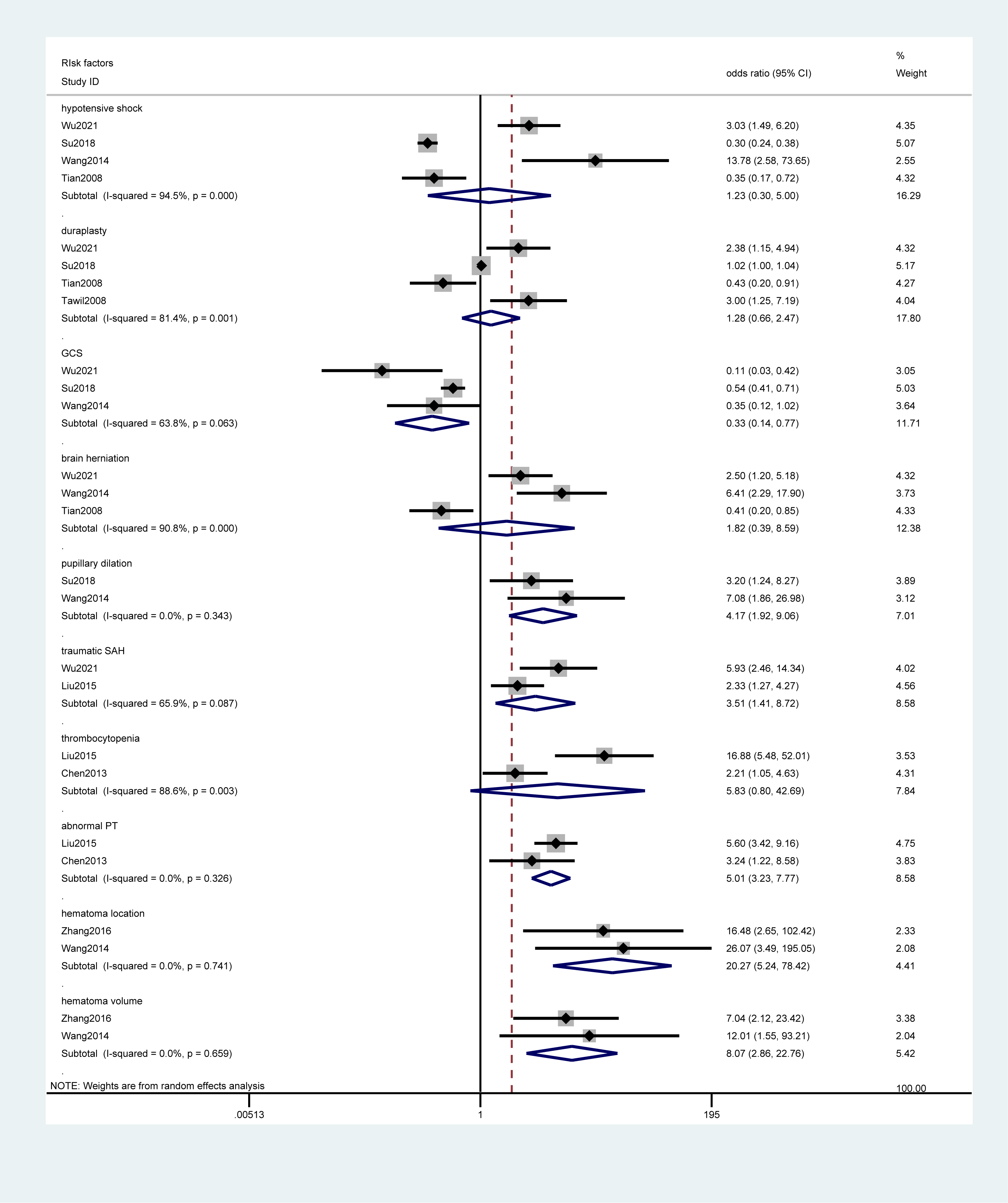

Supplement: Supplemental Material [file KBIE_A_2070999_SM5651.zip › supplementary/Figure S2.tif]
